# The Influence of the ACTN3 R577X Genotype on Performance in Brazilian National-Level Decathlon Athletes: A Pilot Study

**DOI:** 10.3390/cells14110782

**Published:** 2025-05-26

**Authors:** Jose Ricardo de Assis Nunes, Halil Ibrahim Ceylan, Paulo F. de Almeida-Neto, Eugenia Murawska-Ciałowicz, Nicola Luigi Bragazzi, Gilmara Gomes de Assis

**Affiliations:** 1 João Pessoa University Center (UNIPE), João Pessoa 58053-000, PB, Brazil; jranpb@gmail.com; 2 Physical Education and Sports Teaching Department, Faculty of Sports Sciences, Atatürk University, 25240 Erzurum, Turkey; halil.ceylan@atauni.edu.tr; 3 Department of Physical Education, Federal University of Rio Grande do Norte (UFRN), Natal 59078-970, RN, Brazil; paulo.neto.095@ufrn.edu.br; 4 Physiology and Biomechanics Department, Wroclaw University of Health and Sport Sciences, 51-612 Wrocław, Poland; eugenia.murawska-cialowicz@awf.wroc.pl; 5 Laboratory for Industrial and Applied Mathematics (LIAM), Department of Mathematics and Statistics, York University, Toronto, ON M3J 1P3, Canada; 6 Human Nutrition Unit (HNU), Department of Food and Drugs, Medical School, University of Parma, 43125 Parma, Italy; 7 Escola Superior de Desporto e Lazer, Instituto Politécnico de Viana do Castelo, Rua Escola Industrial e Comercial de Nun’Álvares, 4900-347 Viana do Castelo, Portugal; dr.deassisgg@gmail.com

**Keywords:** ACTN3 polymorphism, decathlon, alpha-actinin, sports performance, muscle strength

## Abstract

Background: Decathlon is a multimodality sport that requires the combination of endurance, strength, speed, and agility. A polymorphism present in the gene encoding for alpha-actinin-3 (*ACTN3*) potentially influences sports performance, since this protein is a structural component of skeletal muscle contributing to muscle contraction effectiveness. Aim: To investigate whether the presence of the *ACTN3 R577X* polymorphism is associated with decathlon athletes’ performance in the different modalities of decathlon. Methods: Thirty-one male athletes from the Brazilian national federation of decathlon aged between 18 and 50 years were genotyped for the *ACTN3 R577X* polymorphism using real-time polymerase chain reaction (RT-PCR). The athletes’ latest decathlon performances were recorded over ten competitions. The Hardy–Weinberg equilibrium was verified. Pearson’s correlation coefficient was utilized to assess the relationship between the obtained sports performance (score) by event and sets of events (speed events, jumps, and throws) with significance considered at *p* < 0.05. Results: Strong and significant correlations were identified between the speed events, the jumping, and the launching performances. Among the athletes, the distribution of *ACTN3* genotypes was as follows: *R577R*—51.6%, *R577X*—48.4%, and *X577X*—0%, indicating a complete absence of homozygosity for the non-functional *X* allele in this cohort. No significant differences in sports performance (score) could be observed based on the genotype. Conclusions: Our results may support the importance of the *ACTN3* genotype, specifically, the presence of the *577R* allele, as one of the contributive factors for athletes’ performance in modalities that involve muscle strength, power, and speed. However, given the small sample size and the retrospective nature of this study, further research is warranted.

## 1. Introduction

Alpha-actinin-3 is an actin-binding protein located in the Z-discs of skeletal muscles, where it forms a lattice-like structure and stabilizes the actin filaments within sarcomeres. It is expressed only in fast-twitch skeletal muscle fibers (type II), more specifically in 100% of type IIb and about 50% of type IIa muscle fibers [1]; however, the absence of alpha-actinin-3 does not confer a phenotypic change in the muscle tissue due to a compensatory role of alpha-actinin-2 [2,3].

A common nonsense polymorphism in the gene encoding this protein (*ACTN3*) is found in 18 to 25% of the general population. This polymorphism involves a cytosine to a thymine (C→T) nucleotide substitution, resulting in the replacement of an arginine (R) with a stop codon (X) and the subsequent synthesis of a truncated, non-functional form of alpha-actinin-3 [4,5]. This *ACTN3 R577X* polymorphism has been reported as influencing athletes’ performance in high-intensity and short-duration sports [6]. In addition, a high frequency of the *RX* and *XX* genotypes is present in endurance sports athletes [7]. It has been shown that the *ACNT3 577R* genotype is associated with better performance outcomes in speed, strength, and hypertrophic response adaptations to training, while the *ACNT3 577X* genotype is related to better performance in endurance sports [6,8,9].

Decathlon consists of ten athletics events held over two competition days. It requires a mix of different athletic competencies and skills such as speed, agility, and endurance. The decathlon modalities can be categorized into speed events (100 m dash, 110 m hurdles, and 400 m dash), jumping events (long jump, high jump, and pole vault), throwing events (shot put, discus throw, and javelin throw), and endurance running events (middle-distance—1500 m run).

As previously mentioned, the *ACTN3 R577X* polymorphism has been extensively studied in the context of its influence on physical performance, with evidence suggesting its role in power, speed, and endurance activities. However, despite the physiological demands of decathlon—a sport requiring a unique combination of strength, speed, endurance, and agility—the relationship between this genetic variant and performance across its ten distinct events has not been previously explored. This study is particularly novel because it examines genotypic distributions and their potential impact on diverse athletic modalities within a cohort of national-level decathlon athletes. Understanding these relationships could provide critical insights into how genetic factors contribute to multisport performance, guiding talent identification, training optimization, and athlete development strategies. Additionally, the findings may further elucidate the mechanistic role of the *ACTN3* polymorphism in determining physical capabilities across different athletic contexts, contributing to the broader field of sports genomics. This study, therefore, addresses a significant knowledge gap in the existing body of scholarly literature, offering practical implications for sports science and genetic research. Our aim was to investigate the frequency distribution of the *ACTN3* polymorphism in a sample of national decathlon athletes, exploring whether the genotypes can influence performance in different modalities.

## 2. Materials and Methods

In this cross-sectional descriptive study, 31 male decathlon athletes aged 18 to 50 years were recruited for genotyping according to the following inclusion criteria: (i) having participated in the Brazilian, South American, Ibero-American, World Championship, or the Olympic games, and (ii) reporting at least 10 years of competing experience. Data from the athlete’s scores from 1996 to 2015 were collected from the Troféu Brasil competition in São Paulo, the Brazilian University’s Games (JUBS) in Aracaju City, and the website of the Brazilian Athletics Confederation (Confederação Brasileira de Atletismo, CBA). It is important to highlight that the decathlete’s scores presented in this study represent 58.06% of the top rankings in the Olympic games.

The sample size was determined a priori using the G*Power software, version 3.1. For this purpose, we considered the following inputs: “*t*-tests family,” point biserial correlation model, with an r^2^ of 0.35 (associative explanation of at least 35%) for two-tailed correlations. Accordingly, we adopted α of 0.05 and β of 0.95. As such, the minimum sample size indicated for the present study was 30 subjects.

### 2.1. Ethics

This study was designed according to the guidelines of the Declaration of Helsinki and approved by the Ethical Committee for research involving human subjects of the University Center of João Pessoa (UNIPÊ), following the National Health Council—Resolution 466/2012 of 12 December 2012, CAAE number #45665215.5.0000.5176. All participants were informed about the objectives, procedures, and potential risks involved in this study and provided their consent.

The athletes’ performance was measured by the decathlon score assigned to the tasks. This score reference was taken from the CBA (Table 1 and Table 2).

### 2.2. Sampling and Genotyping

All participants were asked to refrain from eating for two hours before collecting the buccal epithelium. A sterile swab microtube (Netlab, Rio de Janeiro, Brazil) (Eppendorf) was used for this purpose. Immediately before taking the biomaterial, the subjects thoroughly rinsed their mouths with warm water. Samples were separated by centrifugation. The resulting aliquots were placed in a refrigerator and kept until DNA profiling at −18 °C. Genomic DNA was extracted by the Chelex method as described by Walsh et al. [10]. Allelic variants and genotypes of the SNP *rs1815739* (*C577T*) of the *ACTN3* gene on chromosome 11q13.2 were determined in all participants using real-time polymerase chain reaction (RT-PCR) (Applied Biosystems, Waltham, MA, USA). The allelic discrimination was performed using a TaqMan^®^ Pre-Designed SNP Genotyping Assay with fluorescent probes delivered by the validated Assay Id C_590093_1_ (Thermo Fisher Scientific, Waltham, MA, USA), according to the manufacturer’s protocol. In addition, 20% of the DNA samples were tested twice with 100% agreement of the results to ensure the accuracy of PCR diagnostics. Primers were designed according to the Applied Biosystems protocol for the Context Sequence [VIC/FAM]: CAAGGCAACACTGCCCGAGGCTGAC[T/C]GAGAGCGAGGTGCCATCATGGGCAT. The following designations of the *C (R)* and *T (X)* alleles for the *rs1815739* of the *ACTN3* gene were used: homozygous fully functional *CC* genotype (cytosine/cytosine) or *RR* (arginine/arginine); heterozygous (intermediate) *CT* genotype (cytosine/thymine) or *RX* (arginine/stop codon); and homozygous low functional *TT* genotype (thymine/thymine) or *XX* (stop codon/stop codon).

### 2.3. Statistics

Descriptive statistics were performed, including measures of central tendency (mean, standard deviation, and variance), along with respective percentages. The normality of distributions was assessed using the Kolmogorov–Smirnov test, and the homogeneity of the variance was examined through the Levene test. Pearson’s correlation coefficient was utilized to assess the relationship between the obtained sports performance (score) in each event and sets of events (speed events, jumps, and throws). The Hardy–Weinberg equilibrium was verified for describing and predicting the genotype and allele frequency in a given population. Allele frequencies were estimated by counting, and the allele observance was compared to its expected frequency based on the Chi-square statistical test. Differences in sports performance (in points) between genotypic groups (*R577R* vs. *R577X*) were analyzed using the non-parametric Mann–Whitney *U* test. For all inferential data analyses, a significance level of *p* < 0.05 was considered. The data were analyzed using the statistical software SPSS^®^ for Windows, version 23.

## 3. Results

The data from the best scores of this study’s participants, according to the modality’s classification, are shown in Table 3.

Table 4 presents the associations among the athlete’s performances in the decathlon modalities. Performances in the 100 m sprint had shown significant positive correlations with the long jump (r = 0.727, *p* < 0.01), shot put (r = 0.375, *p* < 0.05), high jump (r = 0.526, *p* < 0.01), 400 m sprint (r = 0.759, *p* < 0.01), 110 m hurdles (r = 0.625, *p* < 0.01), pole vault (r = 0.428, *p* < 0.05), and the 1500 m run (r = 0.463, *p* < 0.01), suggesting a shared reliance on speed and power output across these disciplines. The 400 m sprint also exhibited significant correlations with the long jump (r = 0.651, *p* < 0.01), shot put (r = 0.581, *p* < 0.01), high jump (r = 0.583, *p* < 0.01), 110 m hurdles (r = 0.640, *p* < 0.01), discus throw (r = 0.489, *p* < 0.01), pole vault (r = 0.612, *p* < 0.01), and javelin throw (r = 0.500, *p* < 0.01), emphasizing the centrality of anaerobic capacity and total-body coordination in these events. Long jump performance had been significantly related to nearly all other events, including the shot put (r = 0.558, *p* < 0.01), high jump (r = 0.789, *p* < 0.01), 110 m hurdles (r = 0.829, *p* < 0.01), discus throw (r = 0.580, *p* < 0.01), pole vault (r = 0.623, *p* < 0.01), and javelin throw (r = 0.385, *p* < 0.05), with a near-significant trend for the 1500 m run (r = 0.328, *p* = 0.071), reflecting the importance of lower-limb power and agility. Shot put scores had shown highly significant correlations with the high jump (r = 0.583, *p* < 0.01), 110 m hurdles (r = 0.651, *p* < 0.01), discus throw (r = 0.736, *p* < 0.01), pole vault (r = 0.699, *p* < 0.01), and javelin throw (r = 0.640, *p* < 0.01), indicating that strength-oriented events shared overlapping skill sets. Similarly, high jump scores were strongly associated with the 110 m hurdles (r = 0.745, *p* < 0.01), discus throw (r = 0.712, *p* < 0.01), pole vault (r = 0.790, *p* < 0.01), and javelin throw (r = 0.397, *p* < 0.05), suggesting a common neuromuscular profile that favors both vertical power and coordination. The 110 m hurdles stood out as a multidimensional event, significantly correlating with the long jump (r = 0.829, *p* < 0.01), shot put (r = 0.651, *p* < 0.01), high jump (r = 0.745, *p* < 0.01), 400 m sprint (r = 0.640, *p* < 0.01), discus throw (r = 0.675, *p* < 0.01), pole vault (r = 0.607, *p* < 0.01), and javelin throw (r = 0.433, *p* < 0.05), evidencing its reliance on speed, technique, and strength. Discus throw performance correlated significantly with the shot put (r = 0.736, *p* < 0.01), high jump (r = 0.712, *p* < 0.01), 110 m hurdles (r = 0.675, *p* < 0.01), pole vault (r = 0.805, *p* < 0.01), and javelin throw (r = 0.608, *p* < 0.01), reinforcing the technical strength nexus across field events. Pole vault scores demonstrated strong and significant associations with the high jump (r = 0.790, *p* < 0.01), discus throw (r = 0.805, *p* < 0.01), javelin throw (r = 0.546, *p* < 0.01), and shot put (r = 0.699, *p* < 0.01), further highlighting this interconnected cluster. The javelin throw significantly correlated with the shot put (r = 0.640, *p* < 0.01), high jump (r = 0.397, *p* < 0.05), 110 m hurdles (r = 0.433, *p* < 0.05), discus throw (r = 0.608, *p* < 0.01), and pole vault (r = 0.546, *p* < 0.01), but showed no significant relationships with the sprints or the 1500 m run. Finally, the 1500 m run had limited associations, reaching statistical significance only with the 100 m sprint (r = 0.463, *p* < 0.01), whereas its correlations with other events remained weak or nonsignificant, pointing to the relative independence of aerobic endurance performance within the decathlon structure.

Furthermore, the Pearson correlation coefficient between speed and jumps was notably high (r = 0.786, *p* < 0.01), suggesting that athletes with superior sprinting performance tended to excel in jumping events as well. Similarly, the correlation between jumps and launching/throwing was also substantial (r = 0.747, *p* < 0.01), reflecting a shared performance foundation rooted in lower-body power and explosive strength. The association between speed and launching/throwing, while slightly lower (r = 0.634, *p* < 0.01), remained moderately strong, underscoring the interdependence of neuromuscular and biomechanical attributes across these event clusters (Table 5).

In Table 6, the genotypes and allelic frequencies for the entire sample are presented. The results show an equitable distribution of the *RR* (n = 16, 51.6%) and *RX* (n = 15, 48.4%) genotypes, with the absence of the *XX* genotype (0%) and a consequent high allelic frequency for the *R* allele amongst the athletes (0.76). This study’s population adhered to the Hardy–Weinberg equilibrium (*p* = 0.758; q = 0.242; χ^2^ = 3.119, *p* > 0.05).

**Figure 1:**
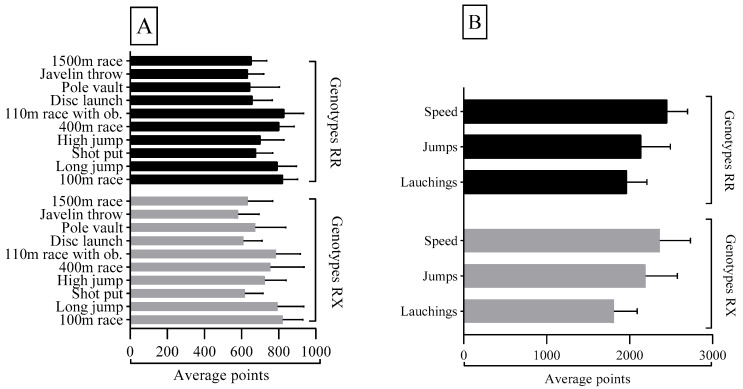
(**A**): Average athletes’ performance per event according to genotypic distribution; (**B**): average sports performance obtained in the sets of speed, jumps, and throws according to the genotypic distribution.

Figure 1A describes the performance points per event according to the genotypic distribution of the *ACTN3 R577X* polymorphism for the entire athlete’s sample. There were no significant differences (*p* > 0.05) between genotypes regarding the athlete’s performance per event. In Figure 1B, we present the performance points per set of events, according to the genotypic distribution of the *ACTN3 R577X* polymorphism for the entire sample. There were no significant differences (*p* > 0.05) in the scores obtained in each set of events between both genotypic groups.

## 4. Discussion

Our results provide a description of the genotypic profile of national competitors of decathlon for the *ACTN3 R577X* polymorphism. Our results show that there is a moderate to strong association between performances in the different events that require speed and agility in decathlon. The sole exception was noticed for the performances in the half-distance race (1500 m), which is an endurance task, that was weakly associated with those of 100 m dash.

Despite the technical specificities that are peculiar to each event, these associations are expected as it is well established that speed and agility are strongly associated with motor skills that rely on muscular strength and power, like jumping and throwing [11,12]. This is due to the rate of force development (RFD) required for performance in expressly explosive motor activities [12], and the RFD is partially determined by innate qualities of the neuromuscular system, which include the biochemical and contractile properties of the muscle fibers [13].

Considering the scores by sets of events, the athletes achieved the highest scores in the speed events (100 m, 400 m, and 110 m hurdles) (total of 2418 points; average of 806.0 points), followed by the jumping events (long jump, high jump, and pole vault) (total of 2188 points; average of 729.0 points), and launching events (shot put, discus, and javelin throw) (total of 1932 points; average of 644 points). The average score in the medium distance run (633.0 points) was the lowest of all events, indicating that the sample of athletes was primarily composed of sprinters.

Analysis of the distribution of the *ACTN3 R577X* polymorphism revealed that 51.6% (n: 16) of athletes were homozygotes for the arginine base (*R577R* genotype) and that 48.4% (n: 15) of athletes presented the stop codon in one of the alleles (*R577X* genotype). The predominance of the *R* allele in athletes specializing in activities requiring explosive muscle contractions and power suggests that individuals expressing the non-polymorphic *ACTN3* isoform possess a morphophysiological advantage in developing muscle power. Our findings are in agreement with the reports of several studies that identified a higher frequency of the *R* allele in strength/power activities, while the predominance of the *X* allele is described in resistance activities [8,14,15].

The available body of scholarly evidence indicates that the *ACTN3* genotype influences muscle phenotypes and physical performance. Yang et al. [6] reported a lower frequency of the *X577X* genotype in speed athletes (8%) compared to endurance athletes (24%) and non-athletes (18%). In contrast to our findings, Niemi and Majamaa [8], who studied Finnish endurance and sprint athletes, found that the frequency of the *ACTN3 X577X* genotype was higher while the frequency of the *R577R* genotype was lower among endurance athletes. Additionally, they pointed out that none of the top Finnish sprinters had the *X577X* genotype. Similarly, the study by Moran et al. [16] of 992 Greek adolescents reported a strong association between the *577R* allele and faster times in a 40 m sprint. In addition, the data of Cieszczyk et al. [17] reported that sprint swimmers, sprint runners, and weightlifters showed a higher frequency of the *577R* allele compared to non-athletes. Nevertheless, this has not been evidenced in all sports. For instance, in a sample of all-time-best Spanish judo male athletes that were compared with ethnically matched nonathletes [18], no differences were found in the allele or genotype distributions, implying that the *R577X* polymorphism was not associated with the status of being an elite judo athlete in the Spanish population.

The *ACTN3 R577X* polymorphism appears associated with several aspects of athletes’ performance, such as strength, speed, and the proportion of muscle fibers—type II (fast) and type I (slow) [3,19,20]. In the human muscle tissue, there are two sarcomeric (calcium-insensitive) alpha-actinin isoforms, encoded by the genes *ACTN2* and *ACTN3*. As previously mentioned, these alpha-actinins mainly compose the sarcomeric Z-line, where they form a lattice structure that anchors together the actin-containing thin filaments to stabilize muscle contraction and interact with a variety of signaling and metabolic pathways. The expression of alpha-actinin-3 is likely restricted to fast glycolytic skeletal muscle fibers, while the alpha-actinin-2 isoform is predominantly present in cardiac and oxidative skeletal muscle fibers [21].

Vincent et al. [22] compared the fiber type composition in the right *vastus lateralis* of *X577X* vs. *R577R* subjects. Individuals who were *R577R* homozygotes showed relatively higher dynamic *quadricep* torques than *X577X* homozygotes. Of note, their fiber type characteristics significantly differed. The percentage surface and number of type IIx fibers were greater in the *vastus lateralis* of *R577R* compared to *X577X* homozygotes and the alpha-actinin-3 protein content was systematically higher in type IIx compared with type IIa fibers (1.17 IIx to IIa ratio). Norman et al. [9] investigated the power and fatigability as well as the expression levels of alpha-actinin-2 and alpha-actinin-3 in the *vastus lateralis* of moderately trained individuals with different *ACTN3* genotypes. Their findings showed no difference in the power output, fatigability, or force-velocity across individuals with different *ACTN3* genotypes. However, training was able to promptly increase the peak torque in *R577R* subjects but not in *X577X* homozygote individuals. Their data revealed that the expression of alpha-actinin-2 is affected by the muscle content of alpha-actinin-3 in a manner that alpha-actinin-2 might compensate for the lack of alpha-actinin-3 and potentially counteract phenotypic consequences. Together, these findings indicate that the *ACTN3 577X* polymorphism influences the mechanistic role of alpha-actinin-3 in generating muscle contraction and, consequently, the capability to develop strength and speed.

It is important to note that sports performance is not solely supported by pure manifestations of strength and power. Also, homozygosity for the *577X* allele might result in the deficiency in the alpha-actinin-3 function but does not necessarily result in an abnormal muscular phenotype [23]. Alternatively, the *577X* has been proposed as a metabolically thrifty allele [17]. All these findings may justify the absence of differences in performances between the genotypic groups *R577R* vs. *R577X* that were exhibited by the decathlon athletes involved in the present study.

Our study is the first study in Brazil presenting data on the relationship between the *ACTN3 R577X* polymorphism and sporting performance in decathlon. Despite the originality and relevance of our findings, this study has some limitations. Firstly, it does not include the genotypic characterization of a non-athlete control group. Secondly, the broad age range of participants in our study necessitated the retrospective use of their best recorded competitive performances over their careers. Consequently, environmental factors such as location, timing within the season, and the age at which these scores were achieved were neither controlled nor adjusted for. Additionally, important confounding variables—including training intensity, volume, coaching quality, and access to facilities—were not measured or adjusted for, potentially influencing both performance outcomes and genotype-expression relationships. Nevertheless, our study contains important merits such as access to high-level multisport athlete’s data. Finally, although an a priori power analysis was conducted, the sample size remains relatively small, limiting the ability to draw robust conclusions and potentially accounting for some of the non-statistically significant results observed.

Despite the limitations mentioned above, the findings of the present study provide valuable insights for coaches, sports scientists, and athletic trainers working with decathlon athletes. By identifying the presence of the *ACTN3 R577X* genotype, it may be possible to tailor training programs to better suit the genetic predispositions of athletes. For instance, athletes with the *R577R* genotype, associated with greater muscle strength and power, could benefit from a focus on explosive power and speed-oriented training to maximize their potential in events such as sprints and jumps. Conversely, athletes with the *RX* genotype, who may have a more balanced strength–endurance profile, could have their training adjusted to improve performance in endurance events like the 1500 m run while maintaining their strength capabilities. Furthermore, the results could aid talent identification programs by providing genetic information that complements traditional performance testing, helping identify athletes with the potential for success in multisport disciplines. Lastly, these insights may also contribute to injury prevention strategies by understanding the role of genotype in muscle adaptation and recovery, optimizing long-term athlete health and performance. For instance, comparing muscle damage (creatine kinase—CK, alpha-actin, and interleukin 6 (IL-6)) in professional soccer players with different *ACTN3* genotypes, Pimenta et al. [24] reported that players who were *X577X* homozygotes presented a higher level of exercise-induced CK and alpha-actin compared to *R577R* and *R577X* athletes. Meanwhile, *R577R* and *R577X* athletes had higher levels of testosterone and IL-6 compared to athletes *X577X*. Their results suggest that *X577X* homozygous athletes are more susceptible to eccentric muscle damage. In the context of decathlon athletes—who are subjected to cumulative mechanical stress across a diverse set of high-intensity, eccentric, and plyometric disciplines—such genetic predispositions could have pronounced implications. It can be hypothesized that athletes with the *X577X* genotype may be at increased risk for overuse injuries and prolonged recovery times due to suboptimal muscular resilience. Consequently, integrating *ACTN3* genotyping into personalized training and recovery frameworks may help inform load management, periodization strategies, and nutritional or pharmacological interventions aimed at safeguarding musculoskeletal integrity across the decathlon’s multifaceted demands.

## 5. Conclusions

Our findings revealed a strong association between speed, jumping, and throwing performances in decathlon athletes, reflecting the complex physiological requirements of this sport. Decathletes demonstrated a superior specialization in speed tasks, which heavily rely on muscular strength and power. The genotypic distribution in our sample showed a frequency of 51.6% for *R577R* homozygous and 48.4% for *R577X* heterozygous athletes, with the absence of the *XX* genotype, aligning with previous research suggesting a predisposition of the *R* allele in power and strength-oriented sports. Although no statistically significant differences could be observed between genotypes in performance outcomes across decathlon events, our results may support the contributive role of the *577R* allele in the development of muscle strength and power, key attributes for high-level performance in speed and jumping events. The lack of performance disparity between genotypes could highlight the multifactorial nature of athletic success in decathlon, where factors such as training, technical proficiency, and environmental influences likely interact with genetic predispositions. This study is the first to investigate the influence of the *ACTN3 R577X* polymorphism on performance in a cohort of national-level decathlon athletes, filling a critical gap in sports genomics research. These findings underscore the complexity of genetic contributions to multisport performance and provide a foundation for future research exploring gene–environment interactions in decathlon and other multisport disciplines. Practical applications of this work include personalized training strategies and talent identification programs that integrate genetic insights with traditional performance metrics, ultimately advancing athlete development and sports science.

## Figures and Tables

**Table 1:** Presents the points per result in day-one decathlon events.

100 m Race		Long Jump		Shot Put		High Jump		400 m Race	
RES-s	Points	RES-m	Points	RES-m	Points	RES-s	Points	RES-s	Points
10.50	975	7.39	908	14.02	730	1.99	794	49.67	830
10.51	973	7.38	905	14.01	729	1.98	785	49.69	829
10.52	970	7.37	903	13.99	728	1.97	776	49.72	828
10.53	968	7.36	900	13.97	727	1.96	767	49.74	827
10.54	966	7.35	898	13.96	726	1.95	758	49.76	826
10.55	963	7.34	896	13.94	725	1.94	749	49.78	825
10.56	961	7.33	893	13.93	724	1.93	740	49.80	824
10.57	959	7.32	891	13.91	723	1.92	731	49.82	823
10.58	956	7.31	888	13.89	722	1.91	723	49.85	822
10.59	954	7.30	886	13.88	721	1.90	714	49.87	821

RES: result; s: seconds; m: meters.

**Table 2:** Presents the points per result in day-two decathlon events.

110 w/Obstacle		Disc Launch		Pole Jump		Javelin Throw		1500 m Race	
RES-s	Points	RES-m	Points	RES-m	Points	RES-m	Points	RES-min	Points
14.20	949	44.16	750	4.79	846	60.12	740	4:39.33	685
14.21	948	44.11	749	4.78	843	60.05	739	4:39.49	684
14.22	946	44.06	748	4.77	840	59.98	738	4:39.65	683
14.23	945	44.02	747	4.76	837	59.92	737	4:39.80	682
14.24	944	43.97	746	4.75	834	59.85	736	4:39.96	681
14.25	942	43.92	745	4.74	831	59.78	735	4:40.12	680
14.26	941	43.87	744	4.73	828	59.72	734	4:40.28	679
14.27	940	43.82	743	4.72	825	59.65	733	4:40.44	678
14.28	939	43.77	742	4.71	822	59.58	732	4:40.60	677
14.29	937	43.72	741	4.70	819	59.52	731	4:40.76	676

RES: result; s: seconds; m: meters; min: minutes.

**Table 3:** The scores (in points) obtained by the athletes in this study.

Set of Events	Sum of Points	Average Points	Fitness Skills
Speed	2.418	806	Explosive strength, displacement speed, agility
Jumps	2.188	729	Travel speed, explosive force
Throwing and Launching	1.932	644	Maximum strength, explosive strength
Medium Distance—1500 m	--	633	Aerobic power

**Table 4:** Pearson correlation coefficient among scores in the decathlon events for the entire sample.

Tests	R. 100 m		L.J	S. Put	H.J	C. 400 m	R.110 m w/H	T. of Disc	Pole Jump	T. of the dart	R. 1500 m
100 m race	r	1	0.727 **	0.375 *	0.526 **	0.759 **	0.625 **	0.336	0.428 *	0.292	0.463 **
	*p*		0	0.038	0.002	0	0	0.064	0.016	0.111	0.009
400 m race	r	0.759 **	0.651 **	0.581 **	0.583 **	1	0.640 **	0.489 **	0.612 **	0.500 **	0.285
	*p*	0	0	0.001	0.001		0	0.005	0	0.004	0.12
Long jump	r	0.727 **	1	0.558 **	0.789 **	0.651 **	0.829 **	0.580 **	0.623 **	0.385 *	0.328
	*p*	0		0.001	0	0	0	0.001	0	0.033	0.071
Shot put	r	0.375 *	0.558 **	1	0.583 **	0.581 **	0.651 **	0.736 **	0.699 **	0.640 **	0.035
	*p*	0.038	0.001		0.001	0.001	0	0	0	0	0.853
High jump	r	0.526 **	0.789 **	0.583 **	1	0.583 **	0.745 **	0.712 **	0.790 **	0.397 *	0.04
	*p*	0.002	0	0.001		0.001	0	0	0	0.027	0.832
110 m race with ob.	r	0.625 **	0.829 **	0.651 **	0.745 **	0.640 **	1	0.675 **	0.607 **	0.433 *	0.309
	*p*	0	0	0	0	0		0	0	0.015	0.09
Disc launch	r	0.336	0.580 **	0.736 **	0.712 **	0.489 **	0.675 **	1	0.805 **	0.608 **	−0.155
	*p*	0.064	0.001	0	0	0.005	0		0	0	0.406
Pole jump	r	0.428 *	0.623 **	0.699 **	0.790 **	0.612 **	0.607 **	0.805 **	1	0.546 **	0.037
	*p*	0.016	0	0	0	0	0	0		0.001	0.842
Javelin throw	r	0.292	0.385 *	0.640 **	0.397 *	0.500 **	0.433 *	0.608 **	0.546 **	1	−0.129
	*p*	0.111	0.033	0	0.027	0.004	0.015	0	0.001		0.49
1500 m race	r	0.463 **	0.328	0.035	0.04	0.285	0.309	−0.155	0.037	−0.129	1
	*p*	0.009	0.071	0.853	0.832	0.12	0.09	0.406	0.842	0.49	

r: correlation coefficient; R = race; L.J. = long jump; S. put = shot put; H.J. = high jump; w/H = with hurdle; T = throw. ** The correlation is significant at the 0.01 level (two-sided). * Correlation is significant at the 0.05 level (two-sided).

**Table 5:** Pearson correlation coefficient and its significance between sets of events for the entire sample.

Set of Events	Variable	Speed	Jumps	Launches
Speed	r	1	0.786	0.634
	*p*-value		<0.01	<0.01
	N	31	31	31
Jumps	r	0.786	1	0.747
	*p*-value	<0.01		<0.01
	N	31	31	31
Launching and Throws	r	0.634	0.747	1
	*p*-value	<0.01	<0.01	
	N	31	31	31

r: correlation coefficient.

**Table 6:** Genotype and allele frequencies observed for the *ACTN3* locus for the entire sample are shown in the table below.

Genotype Frequency			Allele Frequency	
R577R (%)	R577X (%)	X577X (%)	577R (%)	577X (%)
16 (51.6%)	15 (48.4%)	0 (0.0%)	76	24

## Data Availability

The data underlying this article cannot be shared publicly due to privacy or ethical restrictions. The data will be shared at reasonable request to the corresponding author.
